# Time-sensitive clinical concept embeddings learned from large electronic health records

**DOI:** 10.1186/s12911-019-0766-3

**Published:** 2019-04-09

**Authors:** Yang Xiang, Jun Xu, Yuqi Si, Zhiheng Li, Laila Rasmy, Yujia Zhou, Firat Tiryaki, Fang Li, Yaoyun Zhang, Yonghui Wu, Xiaoqian Jiang, Wenjin Jim Zheng, Degui Zhi, Cui Tao, Hua Xu

**Affiliations:** 1 School of Biomedical Informatics, The University of Texas Health Science Center at Houston, Houston, TX USA; 2 School of Computer Science and Technology, Dalian University of Technology, Dalian, China; 3 Department of Health Outcomes & Biomedical Informatics, College of Medicine, University of Florida, Gainesville, FL USA

**Keywords:** Clinical concept embedding, Distributional representation, Time sensitive concept embedding, Electronic medical records, Concept similarity, Predictive modeling

## Abstract

**Background:**

Learning distributional representation of clinical concepts (e.g., diseases, drugs, and labs) is an important research area of deep learning in the medical domain. However, many existing relevant methods do not consider temporal dependencies along the longitudinal sequence of a patient’s records, which may lead to incorrect selection of contexts.

**Methods:**

To address this issue, we extended three popular concept embedding learning methods: word2vec, positive pointwise mutual information (PPMI) and FastText, to consider time-sensitive information. We then trained them on a large electronic health records (EHR) database containing about 50 million patients to generate concept embeddings and evaluated them for both intrinsic evaluations focusing on concept similarity measure and an extrinsic evaluation to assess the use of generated concept embeddings in the task of predicting disease onset.

**Results:**

Our experiments show that embeddings learned from information within one visit (time window zero) improve performance on the concept similarity measure and the FastText algorithm usually had better performance than the other two algorithms. For the predictive modeling task, the optimal result was achieved by word2vec embeddings with a 30-day sliding window.

**Conclusions:**

Considering time constraints are important in training clinical concept embeddings. We expect they can benefit a series of downstream applications.

## Background

Distributional representation learning plays an increasingly essential role in many tasks due to its effectiveness in dimensionality reduction and capability in addressing sparsity issues [1]. A milestone is word embeddings trained on texts [2], which has gained remarkable successes in many natural language processing (NLP) tasks such as text classification [3], machine translation [4], relation extraction [5] and question answering [6]. For healthcare data mining, clinical concepts also contain rich latent semantic relationships like those for words in texts. It is difficult to represent clinical concepts using just one-hot coding, and they should be understood from multiple perspectives according to different scenarios. In recent years, distributional representations of clinical concepts (i.e. clinical concept embeddings) learned automatically from clinical data resources have been explored and proven to be useful for some downstream applications such as predictive modeling [7], patient similarity analysis [8] and relation inference [9].

Among the most relevant researches, Choi et al. learned distributed representations of medical codes (e.g. diagnoses, medications, procedures) from electronic health records (EHRs) and claims data using Skip-gram and applied them to predict future clinical codes and risk groups [10]. Likewise, similar methods were studied and applied in predictive modeling by the same research group [7]. Cui2vec was one of the most recent studies in learning clinical concept embeddings [11], which applied word2vec [1] and Glove [12] on multiple medical resources such as structured claims data, biomedical journal articles and unstructured clinical notes. Cai et al. proposed a model that integrated neural attention mechanism, so as to model the time gaps between consecutive medical events [13]. In this study, we adopted multiple state-of-the-art algorithms and extended them to consider temporal information so that time dependencies are included. The algorithms include word2vec, PPMI-SVD (positive pointwise mutual information-singular value decomposition) [14] and FastText (an extension to word2vec based on subword n-gram) [15]. Among them, FastText was seldom used in other concept learning studies and we think it may help improve the representation abilities of concepts that can be categorized by word ngrams (i.e. prefix for some medical codes). We conduct evaluations on both intrinsic evaluations focusing on concept similarity measure and an extrinsic evaluation to assess the use of generated concept embeddings in the task of predicting disease onset. The experiments show that embeddings learned from information within one visit (time window zero) did improve performance on concept similarity measure and the FastText algorithm usually had better performance than the other two algorithms. For the predictive modeling task, the optimal result was achieved by word2vec (Skip-gram) embeddings with a 30-day sliding window.

Table 1 is a brief summary of these popular clinical concept embedding learning studies. As shown in Table 1, the proposed study here is different from previous studies in several aspects. Firstly, most previous studies have focused on the word2vec method for embedding generation, but we included more other methods such as PPMI-SVD and FastText. Secondly, we evaluated their performance using both intrinsic evaluations of concept similarity and an extrinsic evaluation of disease onset prediction. Although Cui2vec [11] and MCE [13] also focus on time-sensitive embeddings, they do not evaluate the use of generated embeddings in downstream tasks such as predictive modeling. Thirdly, we included time dependency information in distinct ways contrast with Cui2vec and MCE. In Cui2vec, they only considered the time window in the negative sampling phase for word2vec but may still suffer from the time gap problem between concepts, while in MCE, they added a new attention layer on word2vec to model the time information, which introduced more computations. In our method, for word2vec and FastText, we let the algorithm dynamically select context concepts based on time gaps and with only slight modifications towards the original algorithms, and for PPMI-SVD, we segmented the input sequence based on time window before computing the co-occurrence matrix.

**Table 1:** A brief summary of several clinical concept embedding studies. Only the largest database used in the study was listed

Study	Method	Data source	patient size	Time-sensitive	Evaluation strategies
Med2vec [10]	word2vec	EHR/ claims	< 1 million	No	Similarity based on vocabularies, predictive modeling and human assessment
Cui2vec [11]	word2vec, glove	claims	60 million	Only in negative sampling for word2vec	Similarity based on vocabularies and human assessment
MCE [13]	attention- word2vec	EHR	< 2 million	With an attention layer	Similarity based on vocabularies
Ours’	word2vec, PMI^a^, FastText	EHR	50 million	Dynamic input windows	Similarity based on vocabularies, and predictive \modeling

^a^pointwise mutual information

Furthermore, data source is another important factor for concept embedding generation. As it has been reported, claims data and EHR data are different but complementary for answering clinical questions [16]; therefore it is important to study concept embeddings from both data sources. Previous studies have utilized large claims datasets (e.g., over 60 million patients in Cui2vec [11]) for concept embeddings; but the size of EHR dataset used is relatively small (less than 2 million patients). In this study, we trained our concept embeddings using a large EHR dataset with about 50 million patients, with the hope to provide some great resources to the community.

**Fig. 1:**
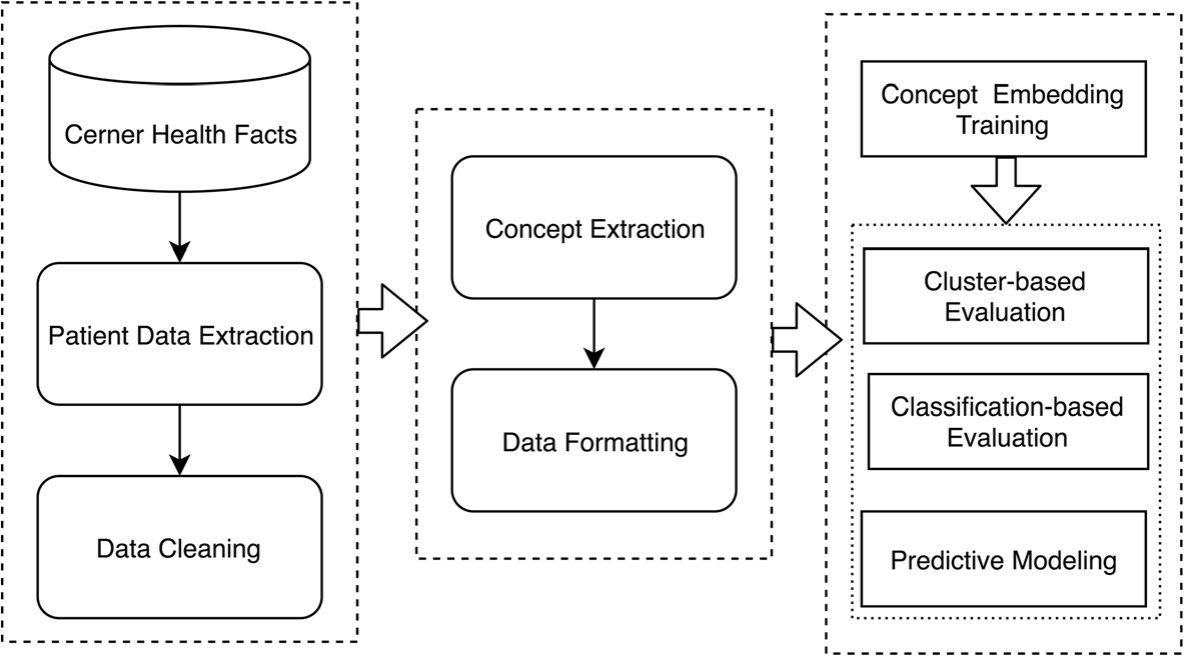
A schematic overview of our approach

To the best of our knowledge, publicly available clinical concept embeddings that are learned from large EHR datasets are still rare [11]. The goal of this study, therefore, is to construct a comprehensive set of clinical concept embeddings by developing different advanced time-sensitive embedding training methods as well as by using extremely large EHR data, and to make these embeddings available to the broader research community. It is expected that the clinical concept embeddings trained on the large EHR dataset can catalyze more downstream applications and meanwhile compensate to existing embeddings trained from other data sources. A schematic overview of this study is given in Fig. 1.

## Methods

### The EHR dataset

Cerner Health Facts® is a database that comprises de-identified EHR data from over 600 participating Cerner client hospitals and clinics in the United States and represents over 50 million unique patients (1995–2015) (https://www.cerner.com/). With this longitudinal, relational database, researchers can analyze detailed sets of de-identified clinical data at the patient level. Types of data available include demographics, encounters, diagnoses, procedures, lab results, medication orders, medication administration, vital signs, microbiology, surgical cases, other clinical observations, and health systems attributes. These clinical data are mapped to the most common standards, for example, most diagnoses are mapped to the International Classification of Diseases (ICD) codes and medications information is in the national drug codes (NDCs).

### Data extraction and pre-processing

We extracted the time-stamped data for all patients from the Cerner database. In this study, we limit our task to generate embeddings for three types of concepts: disease diagnoses (D), medications (M), and procedures (P). All information about D, M, and P are stored chronologically in different tables. Each patient is identified by a unique patient ID, and for each patient’s visit to a health facility, there is a specific visit ID. For each clinical event, a corresponding code is assigned together with its timestamp, indicating when this event happened or stored (i.e. medication information includes prescription time, taken time, and end time, etc.).

**Fig. 2:**
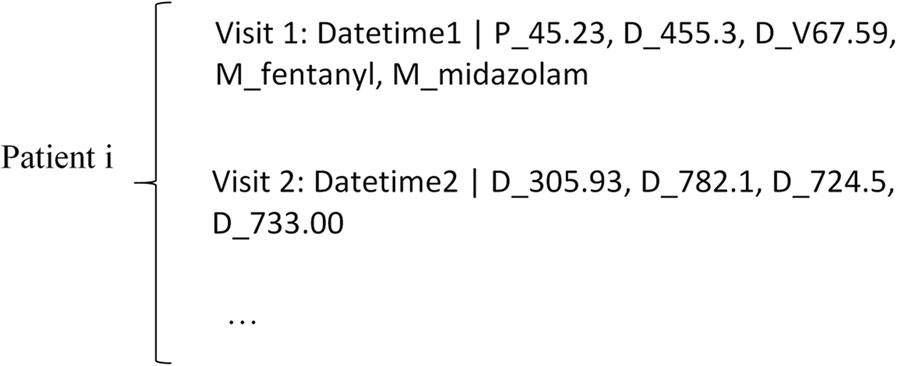
An example showing the data structure for storing the patient information

To facilitate information extraction, we used the following data structure to represent one patient’s records (Fig. 2). In this structure, each patient is identified by a Patient ID (i). The multiple clinical events are distributed in each visit, with distinct Datetime and sorted in an ascending chronologic order. In each visit, the three types of events D/M/P were stored in random orders. We removed the patients containing obvious incorrect information (i.e. with wrong timestamps) in the data cleaning phase and the data were finally stored on the disk in human readable formats for accuracy examination. The disease diagnoses were mapped to ICD-9, medications were normalized to generic names, and for procedures, we used the original Cerner IDs for representation and kept dictionaries that map an ID to ICD-9, HSPCS [17] or CPT4 [18].

### Concept embeddings

Word2vec, as one of the dominators in training distributed representations, has been a routine configuration in many NLP tasks [1, 19]. It trains a two-layer neural networks to reconstruct linguistic contexts of words and each input word is then expanded into a continuous vector. Word2vec can utilize two model architectures to produce distributed representations of words: continuous bag-of-words and Skip-gram, in which Skip-gram performs better in most cases.

In recent years, word2vec has also been applied to learn clinical concept embeddings through feeding it with patients’ medical records [7, 10, 11]. However, most of the existing methods for learning word embeddings lack the consideration of temporal dependencies between adjacent concepts in the modeling stage, which is crucial in the clinical domain and different from language processing. These methods treated the neighborhood events (or visits) equally as adjacent words, and assumed that the events (or visits) in the sliding window reflect the scope of context for prediction (i.e. Med2vec in [10]). Nevertheless, this assumption is not always true, especially when a sequence is sparsely distributed along the timeline. For example, an event *A* happened one year after *B* should be treated differently from *C* happened one day after *B*, which may exist in records of patients who rarely visit a doctor.

Attempts have been made by several researchers in addressing the above issue by including time windows when computing the concept co-occurrence [11] or by adding more neural network layers [13]. In this paper, we tackle this problem by improving three popular word embedding learning methods to time-sensitive versions in a slightly different way: adding time windows on the sequences of input events. Namely, we allow the model to dynamically select the context concepts based on time window during model training.

### Time-sensitive skip-gram model of word2vec

**Fig. 3:**
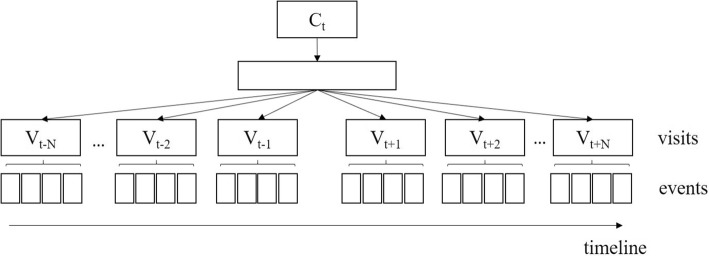
The time-sensitive Skip-gram model for learning clinical concept embeddings

For word2vec, the Skip-gram model was adopted for extensions to learn clinical concept embeddings from structured data. We use the target clinical event to predict its contexts and leverage a dynamic window to define the context scope. In detail, with a target clinical event (concept) C_*t*_ that belongs to the *t*th visit of the patient (*V*_t_), its contextual time window is set to be *N* days before and after the event ([*t-N*, *t + N*]), and the current visit *V*_t_ is also included. Therefore, when we consider *C*_t_, its context concepts include all the possible concepts within the time window. The structure of this time-sensitive Skip-gram model is shown in Fig. 3.

By defining the context window, the equation of Skip-gram can be rewritten into:

1 $$ \frac{1}{\varepsilon}\sum \limits_{t=1}^{\varepsilon}\sum \limits_{C_k\in \left\{{V}_{t-N},\dots, {V}_{t+N}\right\},k\ne t}\log p\left({C}_k|{C}_t\right) $$

where the normalization item $\varepsilon ={\sum}_{i=1}^T{N}_i$, *N*_i_ is the number of distinct clinical events within each visit and *T* is the total number of visits for each patient. And

2 $$ p\left({C}_k|{C}_t\right)=\frac{\exp \left({v}_{C_k}^{\hbox{'}T}{v}_{C_t}\right)}{\sum_{C=1}^V\exp \left({v}_C^{\hbox{'}T}{v}_{C_t}\right)} $$

where *V* denotes the number of concepts in the vocabulary, and *v*_C_ stands for the vector representation of concept *C*.

In this study, we firstly set the window size as 30 days, that is, we consider clinical events happened 15 days before or after the current visit as the context for the target concept. We think that events happened within one month should have much closer relationships with the event of interest. By doing this, we reduce the negative effects from events happened far away from the current timestamp. We also set the time window size to 0 to produce a visit-level embedding matrix, so that only clinical events within the same visit are considered as the context. The Skip-gram model without any time window settings was set as the baseline.

### Time-sensitive PPMI-SVD

Positive Pointwise Mutual Information-Singular Value Decomposition (PPMI-SVD) is a factorization-based method [11, 20]. The connections between clinical events are represented in the form of a co-occurrence matrix *C* in this method. Firstly, the number of times each concept appears inside a window of a particular time duration around the target event is counted. Then, a symmetric PPMI matrix *M* is built based on *C*. Finally, SVD on *M* is performed to get a *USV*^T^ decomposition. The rows of *U* is selected as the embeddings for all concepts in our dictionary.

The PPMI-SVD method has three steps:

Build a co-occurrence matrix *C* with each row/column indexed by a clinical concept. The entry of *C*(*i*, *j*) is the number of times concept *C*_i_ and *C*_j_ co-occur in the same time window.

Build a symmetric PPMI matrix *M* with each row/column indexed by a clinical concept. The entry of $M\left(i,j\right)= PMI\left({C}_i,{C}_j\right)=\log \frac{p\left({C}_i,{C}_j\right)}{p\left({C}_i\right)p\left({C}_j\right)}$, where *p* (*C*_i_, *C*_j_) is the empirical probability of a concept pair appearing within an time window and *p* (*C*_i_) is the marginal probability of *C*. *M*(*i*, *j*) is set to 0 if it is negative.

Obtain concept embeddings by performing SVD on the PPMI matrix *M*.

In this method, we also set the time window of computing the co-occurrence as 0 (visit-level) or 30 days. We did not include a basic version (computing the co-occurrence on patient-level) for PPMI-SVD because timelines for patients vary much.

### Time-sensitive FastText model

FastText is an extension to word2vec in which morphology of words is considered in embedding training. The algorithm of FastText from Skip-gram is by replacing the similarity function *s*(*C*_*v*_, *C*_*t*_) = *C*_*v*_^*T*^ ⋅ *C*_*t*_ to

3 $$ s\left({C}_v,{C}_t\right)=\sum \limits_{z_g\in G\left({C}_v\right)}{z_g}^T\cdot {C}_t $$

where *G* (*C*_v_) is the set of n-grams appearing in *C*_v_ and *z*_g_ is the vector representation for each n-gram *g* (each subword such as *asp*, *spi*,*…* for the word *aspirin*). And then the vector representation of a word can be generated by summarizing the n-grams. By using FastText, the sparsity problem in the representation of rare words can be alleviated using n-grams instead of words. There are also bunches of specific configurations of FastText such as hash map and the selection of *P* (a threshold for cutting off the frequency of words in calculating n-grams), which are used to speed up the training process. Contrast with the previous two methods, FastText largely reduces the training time.

Our intuition of applying FastText is that we assume that the n-gram information can be beneficial for the representation capacity of a clinical concept, such as by modeling prefixes for ICD codes or suffixes for drug names. For time-sensitive settings, we followed the configurations of Skip-gram: one sequence-level without any time window, one visit-level with time window 0 and another 30 days.

### Evaluation and results

We compared the proposed time-sensitive methods with the traditional Skip-gram algorithm with a fixed window size in the evaluation step. Inspired by previous studies (see Table 1), our evaluation plan includes two intrinsic methods on concept similarity: a) clustering-based evaluation; and b) classification-based evaluation; and one extrinsic method: c) predictive modeling-based evaluation. We use the suffix -*baseline* to denote the methods with a fixed length sliding window (5 concepts before and after a specific concept). For the time-sensitive methods, we have a visit-level and a 30-day time window version, with suffixes *-T-visit* and *-T-month*. The three models Skip-gram, PPMI-SVD, and FastText are represented as *SG*, *PPMI*, and *FT* respectively. Dimensions for all the embeddings are set at 200. In total, we have 30,348 distinct concept IDs in the embedding matrix, in which there are 16,418 diagnoses, 11,940 procedures and 1990 medications codes.

### Clustering-base evaluation

Metrics from the clustering theory are adopted for evaluation. The assumption for the clustering-based strategy is that a better concept embedding space should have smaller average distances within each cluster (cohesion), meanwhile have bigger distances between each two clusters (decoupling). The clusters in our current evaluation were based on two existing standard vocabularies, ICD and Clinical Classifications Software (CCS) [21]. They were employed for the evaluation of diagnoses and procedures. For the coding rules in the Cerner database, diagnoses are coded with ICD-9, and procedures are coded with ICD-9, CPT-4 and HSPSC. We selected the concepts with ICD codes as the evaluation set in the current stage.

The in- (cohesion) and out-cluster (decoupling) distances are defined as:

4 $$ {D}_{in}\left(V,G\right)=\frac{1}{\mid V(G)\mid}\sum \limits_{v\in V(G)}\frac{1}{C_{\mid {N}_v\mid}^2}\sum \limits_{\left(u,w\right)\in {C}_v}1\hbox{-} cosine\left(u,w\right) $$

5 $$ {D}_{out}\left(V,G\right)=\frac{1}{C_{\mid V(G)\mid}^2}\sum \limits_i\frac{1}{\mid {N}_i\mid \cdot \mid {N}_j\mid}\sum \limits_{u\in {C}_i,w\in {C}_j,i\ne j}1\hbox{-} cosine\left(u,w\right) $$

where *G* is the pre-defined grouping function such as ICD or CCS, *V*(G) is the whole set of distinct concepts, |*N*_k_| denotes the number of concepts in the *k*th group, and *C*2 N stands for the *2*-permutations of *N*. It is expected that similar concepts would be grouped together based on distance, i.e. ICD codes 493.22 and 493.91 are both with the prefix 493 (*Asthma* in the CCS hierarchy), while concepts describing in different groups, i.e. *Asthma* and *Leukemia*, should have bigger gaps. Based on the above equations, the smaller *D*_in_ is, the better average cohesion is, and the larger *D*_out_ is, the better average decoupling is. The in- and out-cluster distances are shown in Table 2. We tested CCS both on the fine- and coarse-grained level following [9]. The minimum average in-cluster and the maximum out-cluster distances are marked in bold for each column (see Table 2). The best values for in-cluster distances are all generated by FT-T-visit across the three vocabularies, and the values are much smaller than those by other methods, indicating that it can group the codes together with better performance. Compared with different embedding learning methods, we find that FT generally behaves well in in-cluster distances, even for the basic model FT-baseline. On the other side, bigger out-cluster distances are produced by PPMI-T methods, especially PPMI-T-visit, indicating PPMI-SVD has a stronger ability to distinguish different clusters than the other two methods. To summarize, the visit-level embeddings (time window zero) perform better on this evaluation.

**Table 2:** In−/out-cluster distances for different embedding methods on the selected taxonomies

Embedding method	ICD prefix	CCS fine	CCS coarse
SG-baseline	0.1259/0.5925	0.2560/0.7458	0.5730/0.7432
SG-T-visit	0.1172**/**0.5806	0.2438/0.7286	0.5556**/**0.7297
SG-T-month	0.1705/0.6429	0.3074/0.6906	0.5980/0.7115
PPMI-T-visit	0.2028/0.8053	0.3568/0.9530	0.8107/0.9716
PPMI-T-month	0.2032/0.8301	0.3531/0.7158	0.8857/0.9512
FT-baseline	0.0885/0.5543	0.2178/0.6859	0.5446/0.7108
FT-T-visit	0.0687/0.5054	0.2008/0.6732	0.5195/0.6879
FT-T-month	0.0879/0.5521	0.2604/0.7119	0.5664/0.7268

### Classification-based evaluation

The classification-based evaluation is inspired by the Medical Conceptual Similarity Measure (MCSM) proposed by [9]. It is similar to the cluster-based evaluation method except that it uses a K-Nearest Neighbor like algorithm [22] to count how many concepts from the same category will fall in the adjacent area of a given concept based on some similarity criteria (i.e. the cosine similarity). The equation for calculating MCSM is:

6 $$ MCSM\left(V,G,k\right)=\frac{1}{\mid V(G)\mid}\sum \limits_{v\in V(G)}\sum \limits_{i=1}^k\frac{I_G\left(v(i)\right)}{\log_2\left(i+1\right)} $$

where *G* is the pre-defined grouping function such as ICD or CCS, *V*(G) is the whole set of distinct concepts, *I*_G_ is the indicator function, considering whether the *i*th nearest neighbor *v*(*i*) is in the same group as *v* according to the hierarchy of *G*. Generally, the larger MCSM is, the better the embedding method is, since concepts from the same category can be grouped closer. We also used ICD prefix (the prefix before. in ICD-9 codes), CCS fine- (the leaf nodes) and coarse-grained level (cutting off at the 2nd level) as the evaluation standards for MCSM on diagnoses and procedures. The value *K* for calculating nearest neighbors was set at 40 following [9]. The similarities of different methods are shown in Table 3.

**Table 3:** Classification-based similarities for different embedding methods on the selected taxonomies

Embedding method	ICD prefix	CCS fine	CCS coarse
SG-baseline	2.4359	4.3606	8.3281
SG-T-visit	2.3727	4.3016	8.3188
SG-T-month	1.8688	3.6763	7.5117
PPMI-T-visit	2.0986	4.1716	7.9809
PPMI-T-month	1.9313	3.8286	7.5441
FT-baseline	4.7690	6.1711	9.3274
FT-T-visit	5.0215	6.1969	9.3876
FT-T-month	4.5979	5.7873	9.0793

The results in Table 3 demonstrate that FT-T-visit obtains the optimal performance for all the three taxonomies. And similar to the in-cluster similarities shown in Table 2, the FT methods generally behave well on the classification-based evaluations. However, compared with the SG-baseline, other SG methods and the PPMI methods didn’t get satisfying results.

### Predictive modeling task

To further assess the use of such concept embeddings in downstream tasks, we also evaluated it in the context of predictive modeling that is to predict the onset of heart failure, as described in Rasmy et al. [23], where the authors applied a state-of-the-art predictive modeling tool, RETAIN [24], to the task. For convenience, we selected a dataset from one random hospital (Finally we got the data from Hospital #5 in the paper) for this study. The number of patients in the dataset is 42,729, including 5010 cases and 37,719 controls, and the population is also from the Cerner Health Facts® Database. In this experiment, we did not use the RETAIN model because we would like to reduce the effect of the complex model structure to the prediction result. The model used for testing the concept embedding in our work is the basic long short-term memory neural networks (LSTM), which takes all the clinical codes in a sequential order based on their occurrence time, and within each visit, we let the codes keep random.

We tested distinct concept embeddings with and without fine-tuning the embeddings during model training for the heart failure onset prediction task. AUC was reported as the primary evaluation metrics. The ratio of training, development and test set is 7:1:2. The hyper-parameters of LSTM were: batch size = 32, Adam with learning rate 0.01 as the optimizer with decay rate of 0.99, hidden size = 64 for LSTM, and L2 penalty = 0.0001. The AUC values are shown in Table 4.

**Table 4:** AUC values on distinct clinical concept embeddings (values are in %)

Embedding method	Without fine-tuning	With fine-tuning
Randomize	–	83.70
SG-baseline	81.75	84.11
SG-T-visit	81.87	84.29
SG-T-month	82.82	85.42
PPMI-T-visit	79.78	81.01
PPMI-T-month	80.60	82.44
FT-baseline	82.51	84.81
FT-T-visit	82.38	84.69
FT-T-month	82.59	84.88

As shown in the second and third columns of Table 4, we can see that the time-sensitive concept embeddings can generally achieve better results for either with or without fine-tuning, of which the SG-T-month achieved the best performance. The results without fine-tuning can reflect the strengths of the pre-trained embeddings to some extent since the concept representations will not change during training. We see that with good pre-trained representations, the LSTM model can produce reasonable results (around 0.82 on AUC) under these settings. Another baseline for the predictive modeling is the method with randomly initialized embeddings as inputs (Randomize in Table 4), in which the embeddings will be fine-tuned during training. Compared with it, LSTM with most pre-trained embeddings works better, implying that the pre-trained embeddings are helpful to find optimal results in this task. It can also be learned from the results of the time-sensitive methods that in this predictive modeling task, using a 30-day time window for embedding training would have more strong representation capacities settled, compared with methods that with visit-level embeddings. Besides, we consider the most possible reason for the unsatisfying results produced by PPMI-SVD is that it may suffer from the data sparsity problem.

## Discussions

### Visualization by t-SNE

**Fig. 4:**
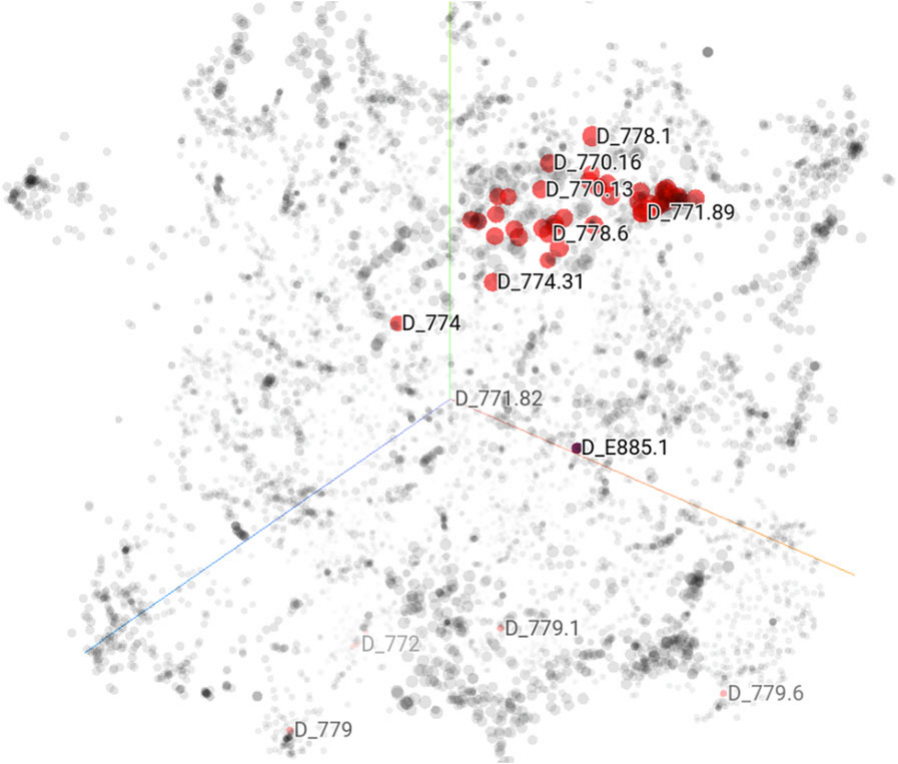
Visualization of the SG-T-month method with t-SNE

To better understand the outcome of the trained concept embeddings, we projected them into a lower-dimensional space and visualized them in the space. t-Distributed Stochastic Neighbor Embedding (t-SNE) is a technique for dimensionality reduction that is particularly well suited for the visualization of high-dimensional datasets [25]. It was employed as the visualization tool for our trained clinical concept embeddings as a qualitative analysis step. To obtain a direct overview of the embeddings, other than the intrinsic and extrinsic evaluations proposed above, we manually queried various prefixes of codes in the t-SNE space to see whether similar concepts could be grouped together. Figure 4 is a screenshot of the t-SNE result based on embeddings of SG-T-month. The highlighted points are with the same ICD prefix 77x for diagnosis (denoting *conditions originating in the perinatal period*).

We notice that several codes belonging to 770.xx (*other respiratory conditions of fetus and newborn*), 774.xx (*other perinatal jaundice*) and 778.xx (*conditions involving the integument and temperature regulation of fetus and newborn*) can be grouped into a neighborhood in the embedding projection space. However, there are also a few codes with the same prefix that cannot be grouped together (i.e. D779), perhaps due to multiple reasons, such as the codes suffer from the data sparsity problem. We have tried multiple other types of concepts on different embeddings and found similar situations. The visualization indicates that similar clinical concepts (or comorbidities) may have a similar context so that they can be frequently co-occurred. For concept embeddings trained by other methods, similar examples can be found. Visualization is just a qualitative analysis strategy, it is difficult to find direct differences between embeddings. However, it might be interesting to explore the differences in surrounding nodes for certain concepts when setting different time windows, which will be part of our future work.

### Clustering- and classification-based evaluations

From the clustering- and classification-based evaluation results shown in Tables 2 and 3, we notice that when evaluating out-cluster similarities (Table 2), PPMI gets an upper hand and when evaluating in-cluster related similarities (including in-cluster similarities from Table 2 and the classification results from Table 3), FTs always get the best performance. These results show that different concept embedding training algorithms behave distinctly based on different evaluation criteria. In addition, we found that another issue that may confused the embedding grouping is that concepts for some comorbidities of certain diseases are likely to be grouped together even they don’t belong to the same category.

Table 5 shows two examples for querying the embedding space of SG-T-month, in which the query ICD-9 code is 789.00 and 401.9 respectively. When querying the code 789.00, most of the top-5 similar codes belong to the 789 category, which are closely related to the specified code. But in the other example, when querying a type of *hypertension*, the most related codes are diverse, from *hyperlipidemia*, *diabetes* to *osteoarthrosis*. These concepts are not under a common sub tree of ICD prefix or CCS, but they are all common diseases for elder patients, likely comorbidity conditions.

**Table 5:** Top-5 relevant concept for two queries based on KNN by embedding SG-T-month

Query	*789.00*	*Abdominal pain, unspecified site*	*401.9*	*Unspecified essential hypertension*
Top-5 results	789.06	Abdominal pain, epigastric	272.4	Other and unspecified hyperlipidemia
	789.09	Abdominal pain, other specified site	250.00	Diabetes mellitus without mention of complication, type II or unspecified type, not stated as uncontrolled
	789.02	Abdominal pain, left upper quadrant	272.0	Pure hypercholesterolemia
	789.07	Abdominal pain, generalized	715.90	Osteoarthrosis, unspecified whether generalized or localized, site unspecified
	787.01	Nausea with vomiting	401.1	Benign essential hypertension

### Predictive modeling task

**Fig. 5:**
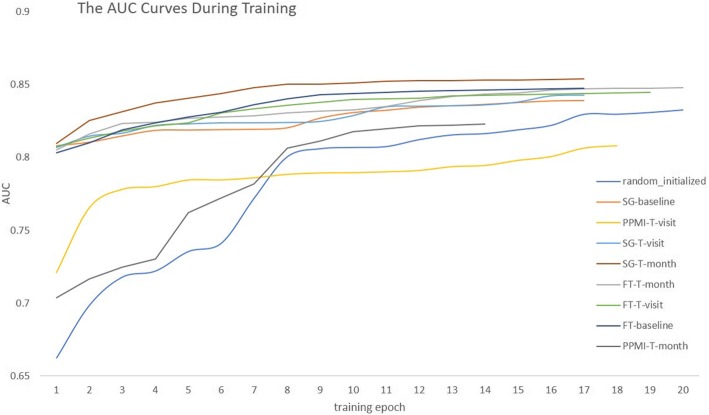
The AUCs on the validation set for each training epoch based on distinct embeddings

Figure 5 shows the AUCs on the validation set during training with embedding fine-tuning. We see that other than the randomly initialized embedding, all pre-trained embeddings behave as expected in that they help the algorithm converge faster. SG-T-month not only gets the optimal AUC point but has the best curve over epochs. SG- and FT-based embeddings can effectively help the algorithm find a better local optimum at the first epoch compared with randomly initialized embeddings and improve the AUC value consistently afterward. However, the PPMI-based embeddings didn’t get satisfying results as others. We consider the most possible reason for the unsatisfying results is that their training processes may suffer from the data sparsity problem.

### Limitation and future work

This study has a couple of limitations. We generated concept embeddings for diseases, procedures, and medications, but did not include lab tests, partially due to that lab test names are not well normalized in the Cerner Health Facts® database. Moreover, for a better usage of the embeddings, a more general normalization of the concepts might need be considered, such as mapping each concept to UMLS CUIs. For the future efforts, we firstly plan to generate concept embeddings for lab tests, by normalizing them according to appropriate ontologies such as LONIC (https://loinc.org/). Secondly, we will further explore the impact of different sizes of time windows. Thirdly, we plan to add more evaluations for downstream tasks such as disambiguation and relation inference.

## Conclusion

In this study, we incorporated time constraints into three popular concept embedding learning models, word2vec, PPMI-SVD, and FastText, and trained the models on a large EHR dataset to construct distinct embedding matrixes. We conducted intrinsic evaluations based on concept similarity measures as well as an extrinsic evaluation of predictive modeling with the trained embeddings and validated the effectiveness of the time-sensitive concept embeddings. The three learning models, however, each has its merits based on different evaluation metrics, indicating that we should select appropriate methods according to specific applications.
